# Design of Performance-Based Concrete Using Sand Reclaimed from Construction and Demolition Waste–Comparative Study of Czechia and India

**DOI:** 10.3390/ma15227873

**Published:** 2022-11-08

**Authors:** Tereza Pavlů, Namratha V. Khanapur, Kristina Fořtová, Diana Mariaková, Bhavna Tripathi, Tarush Chandra, Petr Hájek

**Affiliations:** 1 University Centre for Energy Efficient Building, Czech Technical University in Prague, 27343 Bustehrad, Czech Republic; 2 School of Civil and Chemical Engineering, Manipal University Jaipur, Jaipur 303007, Rajasthan, India; 3 Department of Architecture & Planning, Malaviya National Institute of Technology Jaipur, Jaipur 302017, Rajasthan, India

**Keywords:** fine recycled aggregate, construction and demolition waste, recycled concrete aggregate, recycled masonry aggregate, recycled aggregate concrete

## Abstract

The main goal and novelty of this study is to show the transferability of practices and experiences with the use of reclaimed sand worldwide in the case in two different regions, the Czech Republic and India, which is necessary for both regions due to the sand availability (Czech Republic) and illegal sand mining including criminal offences (India). Due to the deteriorating environmental impacts associated with sand mining, finding substitution possibilities for natural sand is becoming more important worldwide. It is realized that the reuse of construction demolition waste concrete is inevitable in the pursuit of circular concrete and cleaner production, envisioned by the United Nations (UN) as the attainment of ensuring sustainable consumption and production patterns (Sustainable Development Goal 12-SDG 12) with an inclusive approach of partnerships to achieve the goal (Sustainable Development Goal 17-SDG 17) for the validation of results. The basic material properties of reclaimed sand were examined, and its impact on the physical, mechanical, and durability properties of concrete with complete replacement of sand was evaluated. Generally, a slight decline in properties of concrete with fine recycled aggregate was found. No significant decrease was found from usage possibility in the point of view of its utilization in specific structures and conditions. The research shows the slight differences of results between the Czech and Indian investigations, which are not essential for the transferability of the results.

## 1. Introduction

Concrete is the largest consumer of natural resources in the construction sector, which is completely dependent on primary resources, where natural aggregates such as crushed rock, river sand, gravel, cement, and water are most used. Excessive extraction of natural sand (fNA) leads to serious ecological and economic problems, for example, changing the water direction, coastal erosion, building dead-end diversions and holes [1] and, furthermore, illegal sand mining including criminal offences [2]. Due to the concrete demand, the impact of extracting fine natural aggregate (0–4 mm) from rivers and seas becomes essential for finding alternative sources for future concrete production. Besides the ecological and regulatory issues, the production of natural aggregates is related to energy consumption and emission. By the replacement of natural aggregate in concrete mixture with recycled aggregate, the amount of CO_2_-eq. emissions related to the aggregate production decreased from 33 kg to 12 kg [3]. The approach of reusing or recycling the structures that are demolished or renovated is the key factor to achieve circular economy and to attain the targets set for sustainable development goal (SDG) 12 [4,5,6,7,8].

As mentioned before, the substitution of natural sand in concrete production has many advantages. However, it is necessary to mention that the wider use of fine recycled aggregate (fRA) also brings challenges. First, there is high variability of the physical and chemical properties of fRA due to the presence of soil, dust, clay, glass, wood, plastics, etc. Assuming that the pure waste material, concrete or masonry, without unwanted impurities is used for preparing fRA as an aggregate for concrete, the decline of concrete properties becomes acceptable. Even so, if the impurities are removed, there is still a wide range of properties, mostly due to the composition of the parent material, which are dependent on local materials, construction, demolition, and recycling processes’ habits. The fRA particle shape, gradation, specific surface area, and chemical composition of the parent material are the important aspects that lead to inconsistencies in the mechanical properties and durability of mortars and concrete prepared using fRA.

The fRA (<4 mm) can be divided into three types: (1) fine recycled concrete aggregate (fRCA), which originates from waste concrete; (2) fine recycled masonry aggregate (fRMA), which originates from masonry; and (3) fine mixed recycled aggregate (fMRA), which originates from the construction and demolition waste (CDW).

fRCA is produced by crushing waste concrete from CDW [9,10,11,12] and the pre-cast industry [13]. It is made up of natural aggregate particles and old cement paste, mostly attached to the aggregate surface. The decline in properties relates to the old cement mortar. This leads to higher porosity and consequently to higher water absorption and weak interfacial transition zones (ITZ). Furthermore, a higher percentage of fine fraction in fRCA is generally observed with a higher specific surface area [14]. The use of fRCA is usually limited to low-grade applications, such as a filling material for soil stabilization, geosynthetic structures, and road constructions [1]. Furthermore, the fRAC as a substituent for natural sand in cementitious renderings and masonry mortars was verified [15,16,17,18,19,20,21,22]. To allow for more sophisticated applications, there are possible ways to improve the quality of fRCA by removing the old cement mortar by adopting a multistage mechanical process, thermal or chemical treatment, separation using microwaves, or a combination of these processes [23]. On the one hand, these processes could improve the quality of fRCA and provide opportunities for further use of cement paste. On the other hand, it would be more economically and environmentally demanding. In conclusion, optimization of processes and usage is necessary for a meaningful solution to the utilization of fRCA, especially considering the possibilities of practical use in the concrete industry.

fRMA originates from waste masonry and the main constituents are red clay bricks, ceramic, mortar, plaster, and, very often, also waste concrete with aggregate particles and cement mortar. Similarly, with fRCA, the fRMA is more porous compared to natural sand and has a higher water absorption. Generally, the porosity and water absorption of fRMA are higher compared to those of fRCA [24,25,26,27].

For these mentioned reasons, the applications of various fRAs in the concrete industry, due to the unknown origin and properties of parent material, upscaling, and lack of guidelines for testing essential properties such as water absorption influencing the workability and effective water-to-cement ratio, make the practical application extremely challenging.

In contrast to the utilization of coarse fraction for recycled concrete aggregate (RCA) as a substitution of aggregate in concrete, where the investigation has been clearly concluded with the description of all negative effects on the properties of fresh and hardened concrete and durability, such as high porosity and consequently high water absorption and weak interfacial transition zones [1,14,28,29,30], the utilization of fRA has demonstrated inconsistent results. It is reported in previous studies that the use of recycled aggregate (RA) negatively influences the workability of concrete due to its higher water absorption, which consequently leads to a negative effect on mechanical properties, mostly the compressive strength as the key material property of concrete. In the case of fRA, the utilization is more complicated by the fact that the method of measurement of water absorption has not been clearly developed, where the differences between various evaluation methods are huge, and the absorbability of fRA during concrete manufacturing is also not known. Due to these facts, the use of fRA in concrete is quite challenging. Consequently, the standards around the world respond by essentially not allowing the use of fRA (<4 mm), contrary to a coarse fraction, as a possible substitution of natural aggregate in concrete.

The main goal of this study is to evaluate the possibilities of using fRCA as a substitute for natural sand in concrete with an inclusive approach of partnerships between two different regions by following the same research approach. Furthermore, the transferability of practices and experiences is verified. The results, as mentioned in previous studies, of the influence of the recycling technology and properties of the parent concrete on fRA are essential for its future use. For these reasons, the basic material properties of fRA and fine recycled aggregate concrete (fRAC) were examined and compared to find differences in this investigation.

## 2. Background

The use of fRCA [22,31,32,33,34,35,36,37,38,39,40,41] and fRMA [24,25,26,27] as a substitute for natural sand in concrete has been studied by few research groups worldwide.

Nedeljkovic et al. [1] reviewed 171 studies, in which fRCA is proposed to be used for low-grade applications such as cementitious render, masonry mortars, road constructions, soil stabilization, and as a filling material for geosynthetic-reinforced structures, except structural concrete. Studies reveal that the properties of RCA concrete depend on the type and size of the natural aggregate in the parent concrete, the strength of the parent concrete, and the method and number of crushing steps [35,42,43,44,45]. The main complications associated with fRCA are the presence of mortar, a highly angular and irregular shape, and a porous and rough particle structure [31,46,47]. Few studies recommend restricting the replacement of fRCA with NA by up to 30% [31,33,38,46,48,49]. However, to address the problem of CDW, procedures must be developed and researched to administer the full replacement of NA with fRCA. In this regard, few attempts have been made to improve the properties of fRCA concrete (fRCAC) by adopting different approaches to proportion concrete [50,51], control the particle gradation [44], and adjust the method of adding water and the saturation level of fRCA [37,52]. The improvement of the properties of superplasticizers has been also verified [34,53,54].

Evangelista and de Brito [31,37] studied the mechanical and durability properties of concrete with different replacement ratios of natural sand by fRCA. During the mixing procedure, the additional water, which was estimated to be absorbed, was added to the mixture and the effective water-to-cement ratio was estimated. The mechanical behavior was studied for replacement ratios of 10%, 20%, 30%, 50%, and 100% [31]. A slight increase in compressive strength was observed, with a maximum replacement ratio 100%. The mechanical properties (tensile strength and modulus of elasticity) were observed to slightly decrease with increasing replacement ratio with a maximum decrease of 30% of tensile strength and 18% of modulus of elasticity for a 100% replacement rate. The decline of properties for replacement ratios of up to 30% was decided as acceptable. In the case of durability [37], the water absorption by immersion and capillary sorption was observed to increase with increasing replacement ratios. The water absorption by immersion increased to a maximum of 46% and the capillarity sorption increased by 70% for concrete made with 100% fRCA compared to control concrete. On the contrary, it has been found that the depth of carbonation increased by about 110% for the control concrete. In the case of mechanical and durability properties, the authors suggested that 30% is the best replacement ratio for the quality of fRCAC.

Geng et al. [41] investigated the effect of fRCA on the carbonation resistance of fRCAC. Three phenomena were evaluated: (1) the influence of the replacement ratio, which was 20%, 40%, 60%, and 80%; (2) the influence of the different types of fRCA with different fineness moduli; and (3) mixtures with similar workability and different replacement ratios. In this study, the carbonation depth of fRCAC with a 20% replacement ratio was similar to that of the control concrete. As the replacement ratio increased, the increase in the depth of carbonation became evident with the depth of carbonation of concrete with the 80% replacement ratio being 10 times higher than the control mixture. In this study, the influence of the finest particles was also tested. It was found that the fineness modulus also influences the depth of carbonation as a result of the old cement paste contained in the fRCA. Furthermore, the influence of water content was verified, where the workability was improved by adding more water to the concrete mixture; however, the carbonation resistance was reduced drastically. As reported in previous studies [33,55], the higher water-to-cement ratio is expected to increase the porosity of concrete resulting in an increase in the depth of carbonation. In addition, in this study, the workability and compressive strength were also tested. The decreasing workability was attributed to an increasing replacement ratio with a constant water-to-cement ratio; consequently, the decline of compressive strength was more than 50% with an 80% replacement rate. Furthermore, a higher reduction in compressive strength was witnessed by optimizing workability by adding water.

Fan et al. [56] studied the fRCA influence of the crushing method on the properties of fRCAC. Two crushing approaches were compared: (1) one-stage crushing and (2) multistage crushing. It was found that the multistage crushing produces better quality fRCA with a higher density, lower water absorption, lower fineness modulus, and, consequently, a lower impact on the fRCAC properties. The maximum decrease in compressive strength of fRCAC was not more than 20% with the complete replacement of natural sand.

Khatib [36] studied the effect of substitution of natural sand with crushed concrete (fRCA) and crushed bricks (fRMA) for replacement ratios of 0%, 25%, 50%, 75%, and 100%. The amount of cement in the concrete mixture was reduced in proportion to its presence in fRCA. A higher decline of compressive strength (maximum 35%) was witnessed for fRCAC with higher fRCA than that for fRMA. However, in this case, the reduction of compressive strength may be attributed to the lower amount of cement in mixtures.

## 3. Recycling of CDW in the Czech Republic and India

In the case of the Czech Republic, the use of recycled aggregate from construction and demolition waste became increasingly desirable over the last few years. The primary reason is the decreasing amount of available natural resources, which is mostly caused by mining closure and restrictions on the opening of new or expansion of existing quarries, which in turn has escalated the price of natural aggregates. Secondly, owing to the interest in promoting circular practices, demolition and construction companies are increasingly approaching the sorting of individual waste, such as waste concrete and masonry, for on-site use, especially for landscaping. This approach, although not ideal, is satisfactory in many respects, especially if landscaping is necessary on-site. For this reason, the amount of mineral CDW (concrete, bricks, ceramics, etc.) reported as received in a landfill or at a recycling center is relatively small, approximately 4.6 million tonnes per year (2020), which is approximately 450 kg per person, and year-on-year has a declining trend. This means that the amount of CDW reported in landfills and recycling centers is decreasing. On the contrary, the extraction of primary raw materials for the construction industry is still growing and is around 72 million tonnes. For comparison, the extraction of primary raw materials in 2015 was 6400 million kg per person per year, which rose to 6700 million kg per person per year by 2020. As is evident from the statistics compiled annually by the Czech Statistical Office [57], even if there is complete utilization of recycled aggregates from waste, the need for construction aggregates cannot be fulfilled. The Czech Republic covers 4% of the aggregate requirement from concrete and masonry waste; however, if unsorted waste (preferably assuming it gets sorted) is used, it can cover around 7% of the requirement. From the point of view of the requirements of the Czech standard, which corresponds with the EN standard, it is possible to partially replace the coarse fraction in concrete with a coarse fraction of RCA, containing more than 90% of the waste concrete and natural aggregate. The maximum replacement rate is 30% for selected classes of concrete mostly without any environmental burdens. This corresponds to the published results from the investigations carried out worldwide that the replacement of coarse fractions up to 30% does not significantly influence the properties of RAC [52]. However, owing to the problematic quality assurance and water absorption determination, the use of a fine fraction of RCA or RMA, in general, is not allowed by a standard. For all these reasons, it is becoming more and more important to optimize demolition and recycling technology to obtain as many quality materials as possible, which will stop landfilling because natural raw materials are not worth landfilling.

In India, the management and reuse of CDW is a prime concern. A study conducted by the Building Material and Technology Promotion Council [58], New Delhi in the year 2018 indicates that the quantity of CDW in India varies from 1215 to 25–30 million tonnes per year (BMTPC, 2018). The report mentions that the estimated quantity of CDW from new construction is approximately 40–60 kg/m^2^ of the built-up area and that from the demolition of constructed structures is around 300–500 kg/m^2^ of the built-up area. In order to tackle the problem of CDW, recycling plants are set up in a few cities in India. There are four operational recycling plants in India [58]; the first operational large-scale CDW recycling facility was set up in Burari, New Delhi in 2009, followed by another plant in East Kidwai Nagar, New Delhi, and one in Ahmedabad, Gujarat. Most of the other cities have not set up CDW recycling facilities despite having CDW management rules issued by the Ministry of Environment, Forest and Climate Change published by the Central Pollution and Control Board [59]. As per the guidelines, all construction projects and facilities generating more than 20 tons of CDW in a day or 300 tons in a month are identified as bulk generators of CDW and are required to implement a waste management plan from the point of view of the requirements of the Indian standards with the latest revision of guidelines in 2016. The use of RCA as coarse fraction has been permitted up to 50%, 25%, and 100%, respectively, for plain cement concrete, reinforced concrete, and lean concrete with compressive strength less than 15 MPa [60]. Similarly, the use of fRCA is permitted up to 25%, 20%, and 100%, respectively, for plain cement concrete, reinforced concrete with compressive strength less than 25 MPa, and lean concrete with compressive strength less than 15 MPa. On the contrary, the use of RA is not permitted either as coarse or as fine aggregate to produce plain cement concrete and reinforced concrete. RA is allowed for use as coarse aggregate in lean concrete (<15 MPa compressive strength) only.

## 4. Materials and Methods

In total, 13 concrete mixtures (7 from the Czech part and 6 from the Indian part) were manufactured and examined to verify the possible replacement of natural sand by fRA. Two concrete strength classes were chosen for comparison: the concrete class with compressive strength of 20 MPa for plain concrete and the concrete class with compressive strength of 30 MPa for structural (reinforced) concrete. All concrete mixtures contained coarse natural aggregate and recycled fine aggregate. Natural river sand and crushed stone sand (India) in these mixtures were replaced by fine recycled aggregate (fRA) originating from the CDW from waste concrete (fRCA) by both the India and Czech teams and waste masonry (fRMA) by the Czech team. The basic physical properties (density, water absorption), mechanical properties (compressive strength, flexural strength, and modulus of elasticity), and durability (freeze–thaw resistance and carbonation resistance) of concrete were verified and compared.

### 4.1. Fine Recycled Aggregate

As described above, the measurement method of fRA’s density and water absorption has not yet been established, leading to the high differences between published results. Previous studies have reported that the dry density of fRMA ranges from 2000 to 2500 kg/m^3^ and water absorption (WA) ranges from 12% to 15% [24,36,61,62]. The dry densities of fRCA range between 1630 and 2560 kg/m^3^ and WA varies between 2.38% and 19.3% [14,33,34,45,47,63,64,65,66,67]. For comparison, the presented values of the densities of natural sand range between 2530 and 2720 kg/m^3^ and WA for the natural fine aggregate ranged between 0.15 and 4.1%. In conclusion, fRMA and fRCA have lower densities than natural sand [1]. The evaluation methodology for the determination of WA of fRA has not been established, which differs from coarse RA, where the methodology for the evaluation of the properties is clearly defined. This leads to the unclear and non-comparable results presented in available literature, where the measured values differ up to 60% when tested by different operators and methods. Furthermore, it is known that fRA does not absorb water to its full capacity during mixing. As has been published in previous studies, it has been estimated that, during mixing, the amount of water absorbed ranges between 49% and 89% [14]. For this reason, the determination of the effective water-to-cement ratio, which influences the workability and, consequently, the mechanical properties of the RAC, becomes complicated.

**Figure 1:**
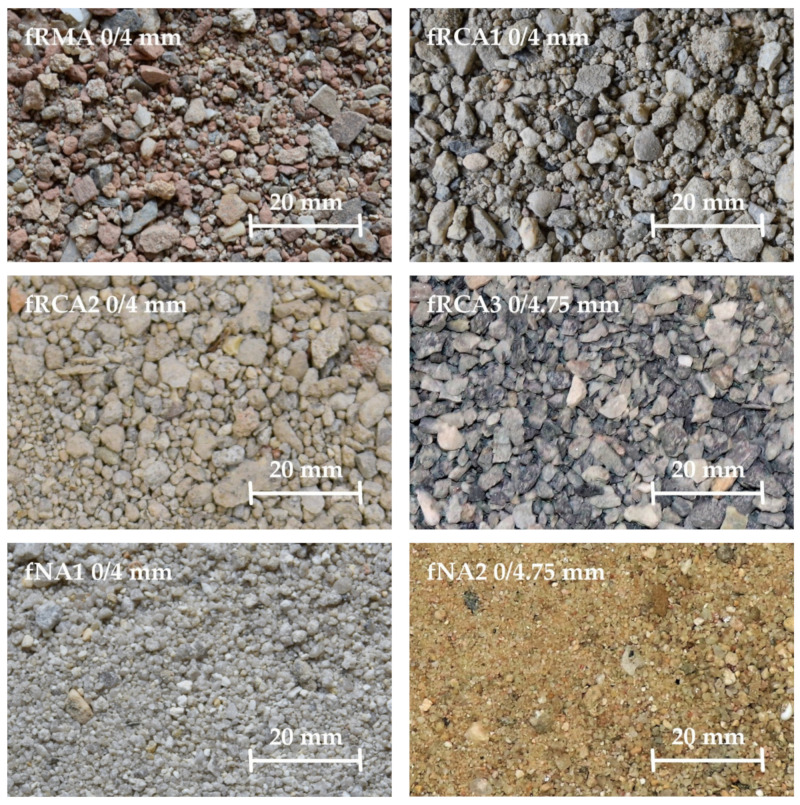

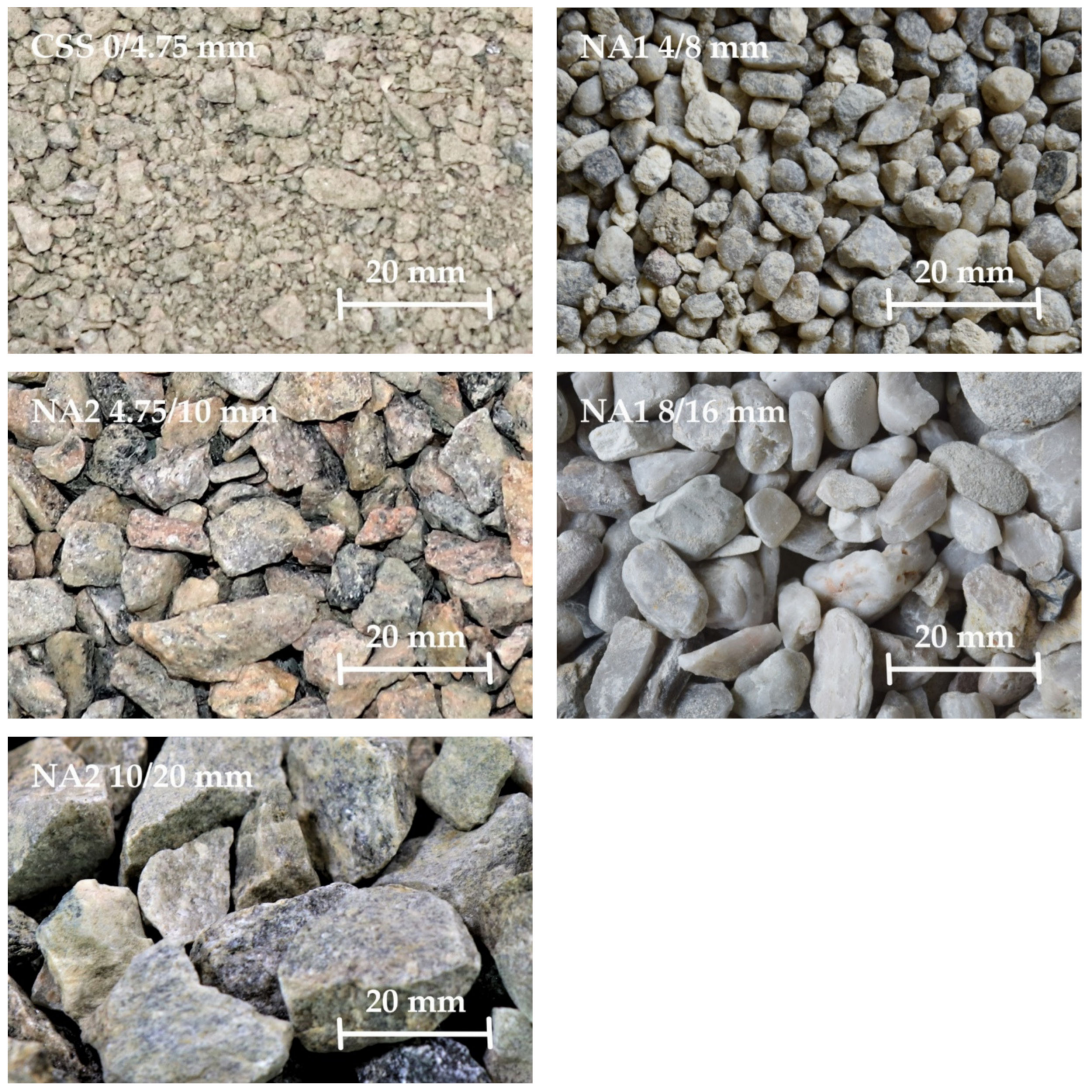
NA and RA used in concrete mixtures.

This study presents the possibility of replacing the whole fine fraction of natural sand (fNA) with fine recycled aggregate (fRA). The Czech team used one type of coarse NA (NA1 fractions 4–8 mm and 8–16 mm) and natural mined sand (fNA1 0–4 mm), two types of fRCA, and one type of fRMA (fractions 0–4 mm), as shown in Figure 1. A type of fRCA1 and fRMA were prepared by a Czech recycling company; the origin of these aggregates was building structures and the aggregate was washed during the recycling process. The other type of fRCA2 was prepared in the laboratory by crushing waste concrete originating from floor structures. For comparison, the India team used one type of coarse NA (NA2 fractions 4.75–10 mm and 10–20 mm), two types of fNA, natural river sand (fNA2) and crushed stone sand (CSS), and one type of fRA (fractions 0–4.75 mm), as shown in Figure 1. fRCA3 was obtained by crushing waste concrete obtained from precast plant set-up for a bridge construction project in Jaipur, India. The waste concrete was crushed in the laboratory and was not washed during the preparation of fRCA3. In this research, the fine fraction of RA (0–4 mm) was used and the coarse fractions were not replaced and remained NA for all mixtures. The main component of fRCA was waste concrete, containing particles of natural aggregate and old cement mortar, and fRMA mainly contained waste masonry (red brick, aerated concrete, and plaster). All tested properties of fRA differed from fNA, especially WA, which was higher and ranged from 3.6 to 8.9% for f RA, while the value for fNA was 1.0% and CSS was 2.8%. This evaluation shows a slightly lower WA of fRMA compared to the results of previous studies [14,33,34,45,47,63,64,65,66,67], which was probably caused by an inconsistent method of measuring fRA WA. The results of fRA WA confirm the conclusions of many previous studies, such as the influence of WA by parent concrete and recycling technology [14]. The lower WA was measured for fRCA1 that originated from normal-strength concrete and was washed during the recycling procedure, so it is assumed that the content of cement mortar was low. In contrast, the higher WA that was measured for fRCA3 may be firstly attributed to the properties of parent concrete, which was high-strength and contained a high amount of cement mortar, and secondly to not washing fRCA3 during preparation. Furthermore, different WA measurement evaluation procedures were used.

**Figure 2:**
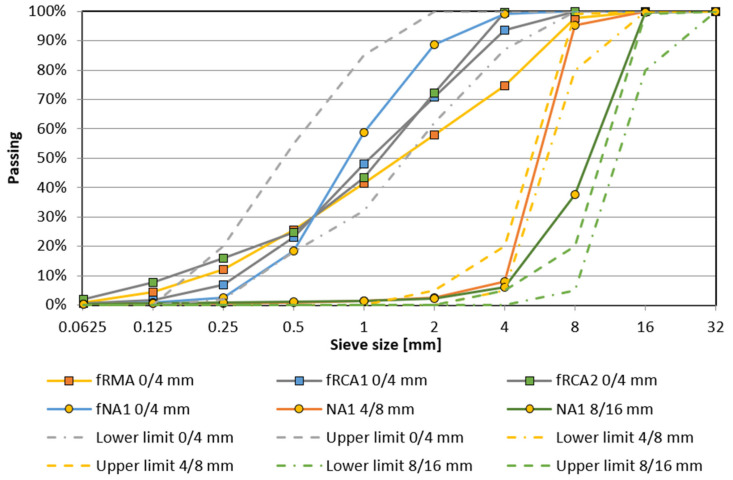
Particle size distribution of aggregates used by the Czech team.

**Figure 3:**
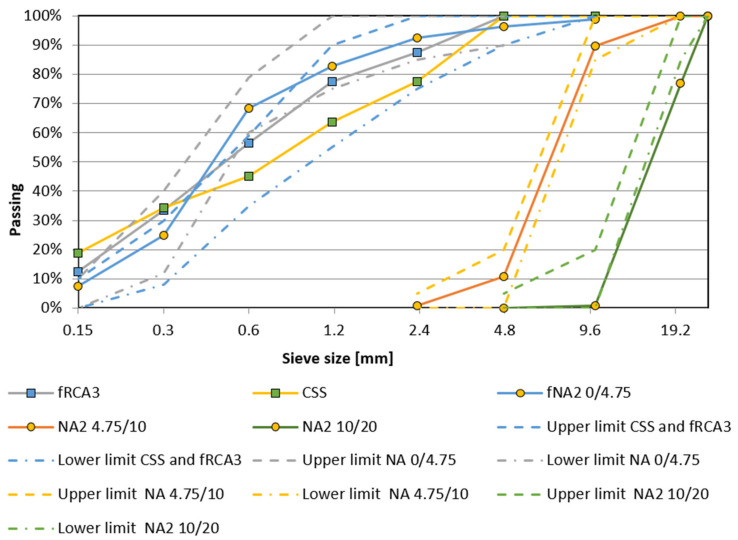
Particle size distribution of aggregates used by the Indian team.

The properties of NA and RA fractions used in this study by both the working teams are given in Table 1 and the valid Czech European standard and Indian standards used for testing the properties are mentioned in Table 2. Figure 2 and Figure 3 present the particle size distributions of all types of NA and RA fractions with limits defined in the European (EN 12620) and Indian standards (BIS:383, 2016) used for preparing concrete mixtures, respectively. The oven-dried particle density of fRA ranges from 2052 kg/m^3^ to 2430 kg/m^3^, where the decline of density in comparison with NA is up to 20%, corresponding with the results of previous studies [14,33,34,45,47,63,64,65,66,67]. Furthermore, fRMA, fRCA1, and fRCA2 do not meet the requirements of the Standard [68] due to difference in granulometry and the presence of fine particles compared to NA (see Table 1 and Figure 2).

### 4.2. Recycled Aggregate Concrete Mixtures 

The 13 concrete mixtures were manufactured for laboratory verification of the properties. The cement CEM I 42.5 R content was 260 kg/m^3^ for the mixtures labelled I and III, 300 kg/m^3^ for II, and 320 kg/m^3^ for IV. The mixture proportions are given in Table 3. Six control mixtures of conventional concrete (NAC IA, NAC IIA, NAC IB, NAC IIB, CSSC IB, and CSSC IIB), three mixtures of strength-class-corresponding compressive strengths of 20 MPa and three mixtures of 30 MPa, respectively, with only NA up to a particle size of 16 mm were produced. In these mixtures, three types of fNA were used: (1) mined sand by the Czech team; (2) river sand; and (3) crushed stone sand by the Indian team. For comparison, in the further 7 mixtures for both concrete classes, the fNA was fully replaced by the different types of fRA: (1) fRCA1, fRCA2, and fRMA by the Czech team and (2) fRCA3 the by Indian team.

The Bolomey particle size distribution curve was used for optimizing the skeleton of the concrete mixtures. The mixing procedure used by the Czech team was similar to that reported by Evangelista and de Brito [65]. Here, a two-stage mixing technique was used, wherein the first stage comprised of 10-minute-long mixing of fRAs with water estimated to be absorbed and 2/3 of the mixing water; subsequently, in the second stage, the remaining constituents were added [1]. The fRAs were mixed with part of the water (water estimated to be absorbed) for 10 min, and after this stage, the remaining constituents and the mixing water were added to the concrete mixture. The water estimated to be absorbed was calculated as a difference between WA of fRA and current levels of aggregate saturation before mixing. The effective water-to-cement ratio for mixtures in the Czech part of the study was estimated as 0.65 for compressive strengths of 20 MPa and 0.55 for 30 MPa reversal. In the case of the Indian part, the effective water-to-cement ratio was estimated as 0.50 and 0.45 for compressive strengths of 20 MPa and 30 MPa, respectively, and a superplasticizer was added to maintain uniform workability of all the concrete mixtures.

### 4.3. Evaluation Methodology

The physical, mechanical, and durability properties were examined by both teams. The dimensions of specimens and testing standards used in the experimental work are shown in Table 4. Testing procedures were designed to be as similar as possible to regional habits. However, the test procedures and their differences are described below. At the age of 28 days, physical and mechanical properties were verified according to Czech and Indian standards. Furthermore, durability (freeze–thaw resistance and accelerated ageing due to CO_2_) and long-term strength development (at the ages of 90 and 180 days) were tested.

The water absorption capacity by immersion, which describes the transport behavior of the material, was obtained on 100 × 100 × 100 mm cube specimens. The specimens were immersed in a water chamber until constant weight and thereafter dried in an oven at 105 ± 2 °C until constant weight. The sorptivity of the concrete specimens of size 100 × 100 × 100 mm with time was determined by conditioning the samples at 105 °C in an oven until their weight stabilized. The specimens were placed on a support device by exposing one of the surfaces to water. The change in mass of specimens was noted at intervals of 0, 1, 10, 30, 60, 120, 240, 1440, 2160, and 4320 min. The slope of the line obtained by plotting absorption against the square root of time gives the sorptivity of the concrete as per ASTM C1585-20.

Prismatic samples of 100 × 100 × 400 mm and 100 × 100 × 500 mm, respectively, cured in water for 28 days were used for assessing the freeze–thaw resistance. The frost resistance was measured according to ČSN 73 1322 (1969) and ASTM C666/C666M-15 (2015) for the Czech team and Indian teams, respectively. Both the teams followed testing by cyclic freezing and thawing at temperatures ranging from −15 °C to +20 °C, where one cycle takes 4 h of freezing and 2 h of thawing. The Czech specimens were subjected to rapid freezing in air and thawing in water and the Indian specimens were subjected to rapid freezing and thawing in water as per ASTM C666/C666M-15 (2015) procedure A. The freeze–thaw resistance was observed for a total of 100 cycles. The measuring of the dynamic modulus of elasticity was performed by the Czech team according to EN 12504-4 (2005) after each phase and by the Indian team according to BIS 13311-1 (1992) after the completion of a total of 100 cycles. The flexural strength was tested by both teams after 100 cycles according to EN 12390-5 (2009) and BIS 516 (1956), respectively, by the Czech and Indian teams.

Prismatic samples of 100 × 100 × 400 mm were stored in a constant laboratory environment for measuring the carbonation resistance. Half of the samples (100 × 100 × 200 mm) were placed for 28 days in laboratory equipment with CO_2_ atmosphere CO2CELL (MMM group) with a concentration of CO_2_ 3.0 ± 0.2%. This test has been prepossessed by the standard ČSN EN 12390-12. Nevertheless, the testing process has been slightly modified and does not fully comply with the standard regulation. The pH drop in the concrete due to CO_2_ was evaluated using the phenolphthalein indication method. In contrast, the accelerated CO_2_ ageing test by the Indian team was performed on samples of different dimensions and in a slightly different way. The depth of carbonation in the concrete cube of size 100 × 100 × 100 mm was tested as per RILEM CPC-18. The samples were cut into four pieces of size 50 × 50 × 100 mm and air-dried for 14 days. The longitudinal sides were coated with epoxy paint and kept in a carbonation chamber at a condition of 3 ± 0.1% CO_2_, 25 ± 2 °C, and 60 ± 5% relative humidity for 28 days. The depth of carbonation was determined by spraying the phenolphthalein indicator on the split surface of the sample.

## 5. Results and Discussion 

In this section, the results of physical, mechanical, and durability properties examined by both the research groups are presented and compared.

### 5.1. Physical Properties

**Figure 4:**
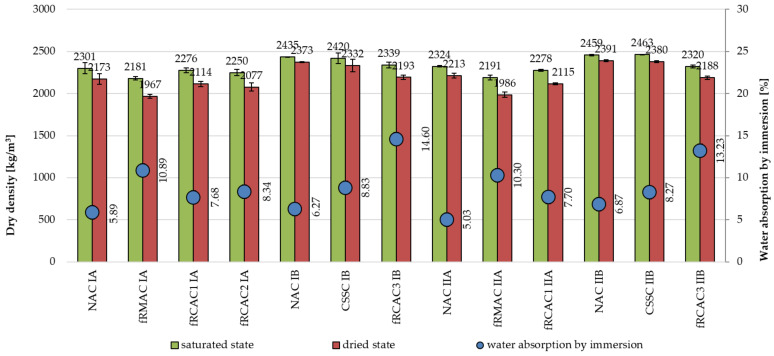
Physical properties of concrete mixtures—saturated surface dry density, oven dry density. and water absorption by immersion.

As reported in previous studies [69,70], the durability of the concrete is fundamentally influenced by its porosity and water absorption. Therefore, immersion-based water absorption and capillary water absorption were evaluated to determine their impact on durability properties. The porosity of concrete and, consequently, the proportion of water with fRCA increased with the increasing replacement level of fRCA [41,71]. The dry density and water absorption of different mixtures are shown in Table 5 and Figure 4. The slight decline in density of RAC in comparison with NAC can be observed for samples in both regions, with the highest decline of about 10%. In general, the water absorption of fRA concrete by immersion was found to be higher than the control mixtures, which corresponds to previous studies [33,37]. As was concluded in many previous studies, water absorption of fRCA and fRMA decreases due to the presence of old mortar. Slight differences between the mixtures developed by the Czech Republic and India were observed. The maximum increase of water absorption by immersion for mixture FRCAC3 IB, manufactured by the Indian team, was more than twice the control concrete. In the case of the Czech team, the maximum increase of 85% was noticed in the fRMAC IA due to the high porous materials, such as red clay bricks, aerated concrete, and mortar, contained in the fRMA. The water absorption by immersion of the fCRAC mixtures increased between 30% and 50%, which is slightly higher than the values presented in previous studies, where water absorption by immersion has been reported to increase from 15% [71,72] to 46% [37] for concrete with the complete substitution of natural sand by fRCA.

Although previous studies have reported a significant increase in capillary absorption from 46% to 95% for 100% fRCAC [53], this was not confirmed in this study. The capillary water absorption measured by both teams was lower than that of the control concretes. The only increase was found for fRMAC IA, which was 44%. On the contrary, the lower decrease was measured for fRMAC IIA with a decline of more than 60%. This decrease may be due to the filling of pores present in the concrete by the products of hydration of unhydrated cement present in the fRCA and, moreover, the water contained soaked in the concrete after curing due to the high WA of the fRA.

### 5.2. Mechanical Properties

#### 5.2.1. Compressive Strength

**Figure 5:**
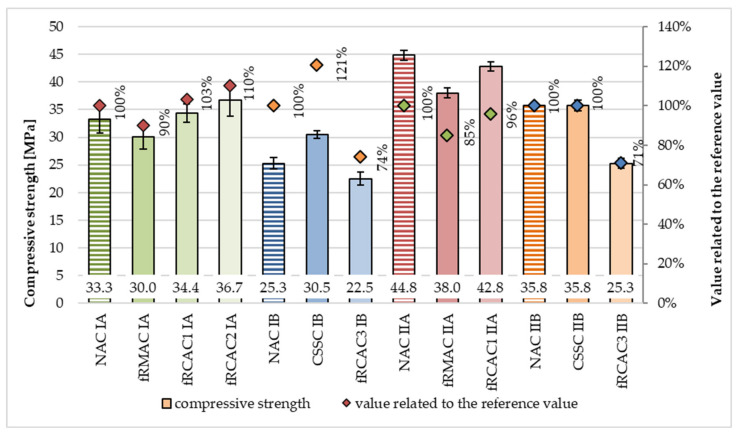
Comparison of compressive strength at 28 days of concrete containing fNA, fRCA, and fRMA with respect to control mixtures.

**Figure 6:**
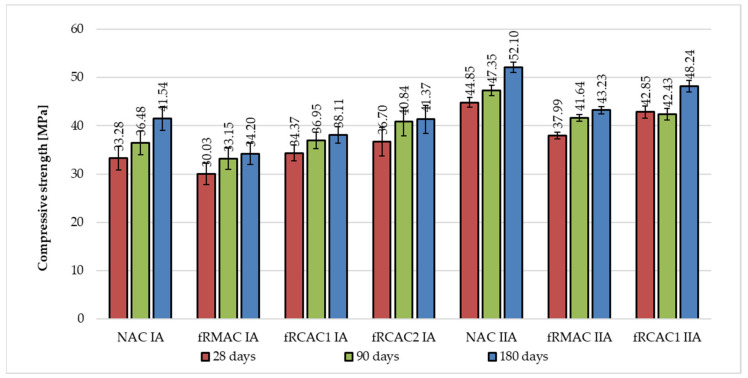
Comparison of long-term development of compressive strength of concrete mixtures prepared by the Czech research group.

**Figure 7:**
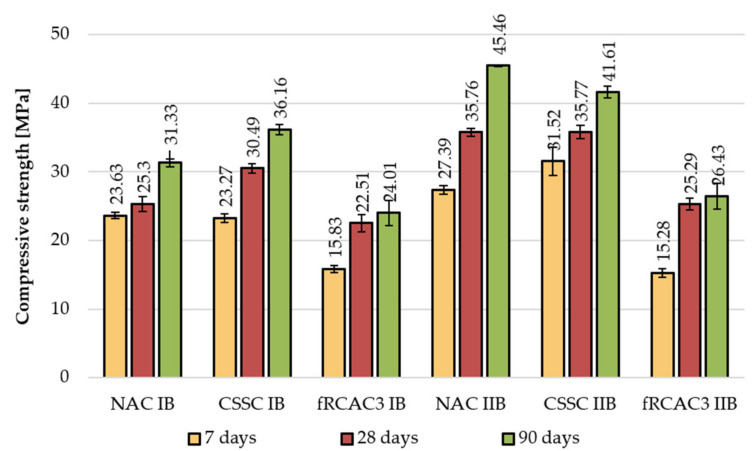
Comparison of long-term development of compressive strength of concrete mixtures prepared by the Indian research group.

The results of compressive strength, which is the key mechanical property of concrete, are presented in Table 6 and Table 7, and Figure 5, Figure 6 and Figure 7. It was observed that the compressive strength of fRCAC differs from previous studies. A study reported higher than control compressive strength [31], and few others reported a similar or lower [31,34,73,74,75] than control concrete. Generally, the sensitivity of compressive strength to the high replacement level of fRCA (100%) has been found, regardless of the strength class of concrete, mostly due to its inaccurately measured water absorption and unknown rate of water during the mixing procedure. For this reason, additional water is used to compensate for these two factors, which leads to the unknown effective water-to-cement ratio, which is only estimated in the case of fRAC. Despite these factors, the compressive strength could be positively affected by the filler effect of fRA, where the finest particles fill the pores and make the structure of the concrete denser, decrease internal tension, and early stress propagation. Moreover, the positive influence on mechanical properties could have an additional internal cure caused by the water absorbed in the aggregate. Furthermore, the fRA particles could have a better interlock between particles due to the rough surface and angular shape [1].

Figure 5 shows the compressive strength of concrete mixtures prepared by both the Czech and Indian teams. Similar to the previous findings, heterogeneous results for compressive strength were observed in this study. In the case of the Czech team, the compressive strength increased (equal to 10%) for concretes containing fRCA1 and 2 compared with control mixtures for lower concrete strength classes. On the contrary, the compressive strength of the mixture with fRCA1 in the higher strength class slightly decreased (4%). Furthermore, the compressive strengths of both fRCA3-containing mixtures were found to decrease in comparison with both control mixes, and, furthermore, the decline was greater compared to the fRCA1 and fRCA2 mixtures. This may be attributed to the difference in source and properties of fRCA3, as it was prepared in India from parent concrete consisting of crushed sandstone. 

At 28 days, the strength of concrete fRCAC3 IB was observed to decrease with respect to the two controls, by 11% with respect to NAC IB and 26% with respect to CSSC IB. The strength of fRCAC3 IIB concrete was found to reduce by 29% in comparison with control NAC IIB and 29% with respect to CSSC IIB. The compressive strength of both concrete strength classes with fRMA slightly decreased (10% and 15%, respectively) compared to the control mix. Furthermore, the development of the compressive strength over time shows a higher rising of fRAC than control concrete.

The decrease in strength and differences between each mixture are probably caused by the presence of an undefined amount of adhered mortar and the amount of additional water to compensate for the higher water absorption and the ability of fRA to soak water during mixing. As previously written, the amount of cement mortar is influenced by the parent concrete and the recycling procedure [14]. In this case, it is assumed that in fRCA1 and fRMA the content of fines was reduced by washing. In contrast, fRCA3 originated from high-strength concrete, so the high amount of cement paste is assumed in the parent concrete and, consequently, the high fine content. In this case, the study confirms previous studies in which the negative effect due to lack of knowledge about fine particles and their influence on the effective water-to-cement ratio was described many times [1]. The maximum replacement rate in the case of compressive strength was stated as 30% [31,33,38,46,48,49].

The development of compressive strength was measured at 28, 90, and 180 days by the Czech team and 7, 28, and 90 days by the Indian team, respectively. The results show a lower increase for fRA mixtures in comparison with the references. The increase of the control concrete was maximally 25% at 180 days; however, in the case of fRA, the maximal increase was 16% (see Figure 6 and Figure 7).

#### 5.2.2. Flexural Strength

Past studies have reported a decrease in the flexural strength of fRCA concrete/mortar with the increase in the fRCA content [19,76]. The maximum reduction in tensile strength was 33% for concrete with a full replacement ratio. As it has been reported, the tensile strength declined with the increasing replacement ratio of natural sand with fRCA and with the water-to-cement ratio increase [77]. In contrast, the flexural strength of mortar at 28 days was found to be higher than the control by 13.7% [44]. However, the maximum replacement ratio in this case was 20% [78]. 

**Figure 8:**
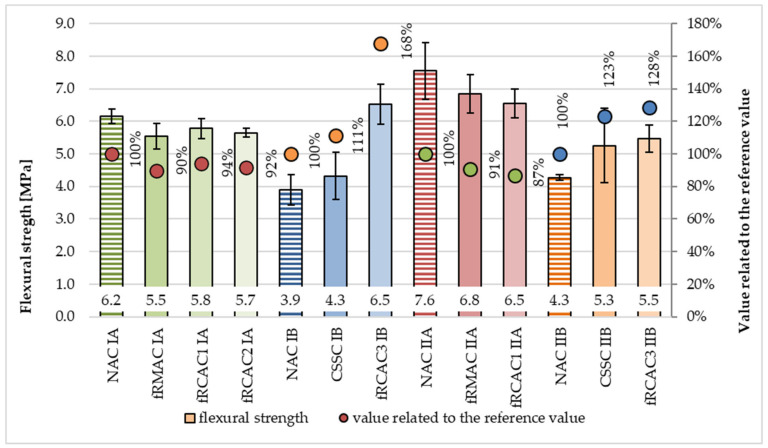
Comparison of flexural strength at age 28 days of concrete containing fNA, fRCA, and fRMA with respect to control mixtures.

The comparison of the flexural strength of concrete mixtures at 28 days is presented in Figure 8. The flexural strength was observed to decrease for all concrete mixtures prepared by the Czech research group. The decrease in properties of the fRAC I mixtures ranged from 6% to 11% and the reduction for fRAC II mixtures was between 11% and 15%. Unexpectedly, in the case of flexural strength, the fRMAC achieved lower declines than both fRCACs. On the contrary, the flexural strength of fRAC mixtures was prepared by the Indian research group. The flexural strength of fRCAC3 IB was observed to increase by 67% and 51% compared with NAC IB and CSSC IB, respectively. The strength of fRCAC3 IIB was observed to increase by 28% and 4% compared with NAC IIB and CSSC IIB, respectively. The higher strength may be attributed to the better interlocking of the fRCA with the paste because of the presence of the uneven surfaces of fRCA.

#### 5.2.3. Modulus of Elasticity

The static modulus of elasticity is the key characteristic for assessing the behavior of reinforced concrete structural elements. Past results of concrete containing coarse RA have reported that, among all the concrete properties, the modulus of elasticity was degraded the highest. The reductions in the static moduli of concretes where natural sand was replaced by fRA range between 9.5% and 17% [34,79]. Wang et al. [80] described that concrete with coarse NA and 100% fRCA showed a reduction in elastic modulus of 5.6–13.5%. Furthermore, a significant decline in modulus of elasticity was reported for low substitution levels (<30%) [81,82]. 

**Figure 9:**
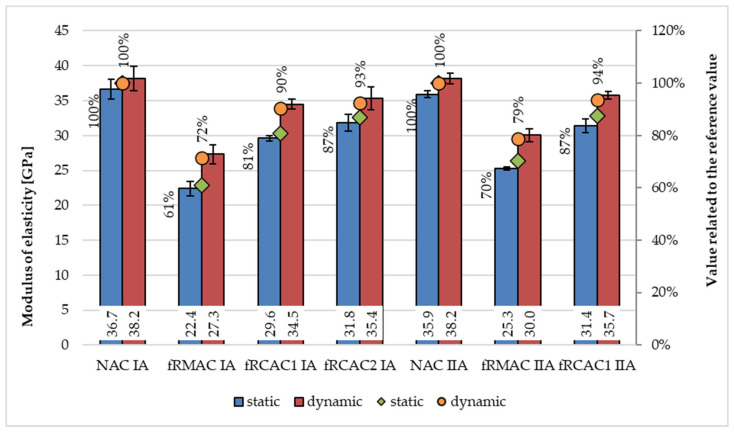
Comparison of static and dynamic moduli of elasticity at age 28 days for concrete mixtures prepared by the Czech team.

**Figure 10:**
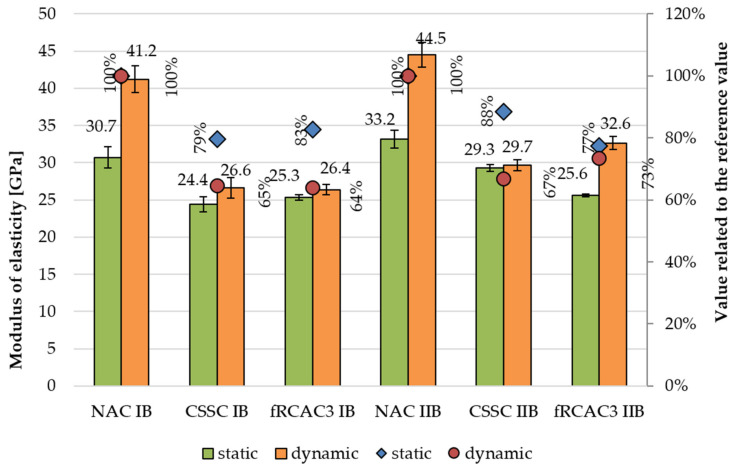
Comparison of static and dynamic moduli of elasticity at age 28 days for concrete mixtures prepared by the Indian team.

The static and dynamic elastic moduli at 28 days of concrete prepared by the Czech and Indian teams, respectively is shown on Figure 9 and Figure 10, respectively. The results show a similar phenomenon for concretes prepared in both regions corresponding with the previous studies. The results confirmed the decrease in static modulus of elasticity, which ranges from 13% to 39% with respect to fRAC I and up to 13% and 30% with respect to fRAC II. The dynamic modulus of elasticity decline was slightly lower and varied between 10% and 28% for fRAC I and between 6% and 21% for fRAC I. Similarly, lower static and dynamic elastic modulus for fRMA concrete for both classes of concrete were observed. The static modulus of elasticity of fRCAC3 IB was observed to decrease by 18% with respect to NAC IB and was similar to CSSC IB. The static modulus of fRCAC3 IIB decreased by 22% and 12% with respect to NAC IIB and CSSC IIB, respectively. The dynamic modulus of elasticity of fRCAC3 IB decreased by 36% with respect to NAC IB and was similar to CSSC IB. The dynamic modulus of elasticity of fRCAC3 IIB was found to decrease by 23% in comparison with NAC IIB and by 10% in comparison with CSSC IIB. The decrease may be attributed to the loss of mortar stiffness due to the presence of adhered mortar in fRCA. Similar findings were observed by past researchers [83].

### 5.3. Durability Properties

The most important factor that affects durability is concrete permeability, which is studied by the oxygen and water permeability test, water absorption by immersion, and capillarity [37,53,71,84,85,86,87,88]. In this study, water absorption by immersion, capillarity, freeze–thaw resistance, and carbonation of concrete containing fRA were verified.

The frost resistance coefficient is according to the Czech Standard established from the flexural strength and dynamic elastic modulus before and after freezing and thawing cycles, respectively.

#### 5.3.1. Freeze–Thaw Resistance

**Figure 11:**
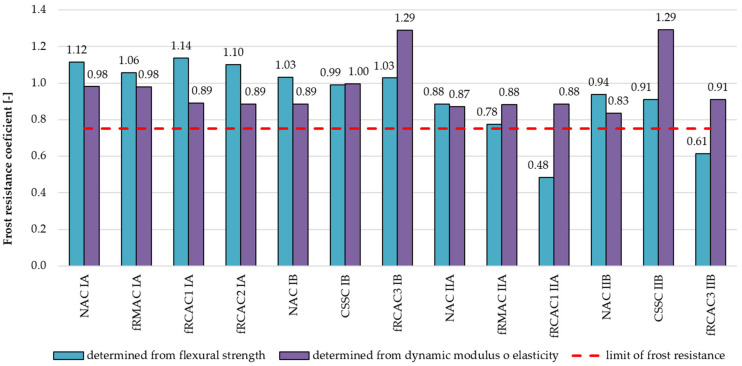
Comparison of frost resistant coefficient calculated from flexural strength and dynamic modulus of elasticity of concrete containing fNA, fRCA, and fRMA measured before and after 100 freeze–thaw cycles.

In the case of freeze–thaw resistance, the positive effect of fRA in the mixture has been found. This phenomenon is caused by the higher porosity of the fRCA, which can provide better hydraulic pressure dissipation. The negative influence of the affecting freezing and thawing could be observed on the fRAC surface, due to the less-resistant mortar, however, without loss of mechanical properties [76,78,89]. This investigation achieved the same results; the freeze–thaw resistance of all examined fRA concretes was similar to or slightly better than control concretes as depicted by the flexural strength, which was measured before and after freezing and thawing (see Table 8). In the case of the dynamic modulus of elasticity (Table 9), a slight decline of the frost resistance coefficient was observed with a maximum 13% reduction of the frost resistance coefficient with respect to control concretes. However, all mixtures conformed to the requirements defined in the Czech national standard, where the frost resistance coefficient must not decrease by more than 25% (see Figure 11). Additionally, the weight and dimensions of the fRCA specimens subjected to 100 freeze–thaw cycles were not significantly altered. The test procedure implemented by the Indian team was slightly different from the Czech team, however, similar results were achieved. The flexural strength of fRCAC3 IB after completion of 100 cycles was comparable with the controls. However, a decrease of 16% and 30% in the flexural strength of fRCA3 II was observed with respect to NAC IIB and CSSC IIB, respectively. The dynamic modulus of elasticity of fRCA concrete after 100 cycles was comparable with controls. These results are in accord with the previous findings [76].

#### 5.3.2. Carbonation Resistance

**Figure 12:**
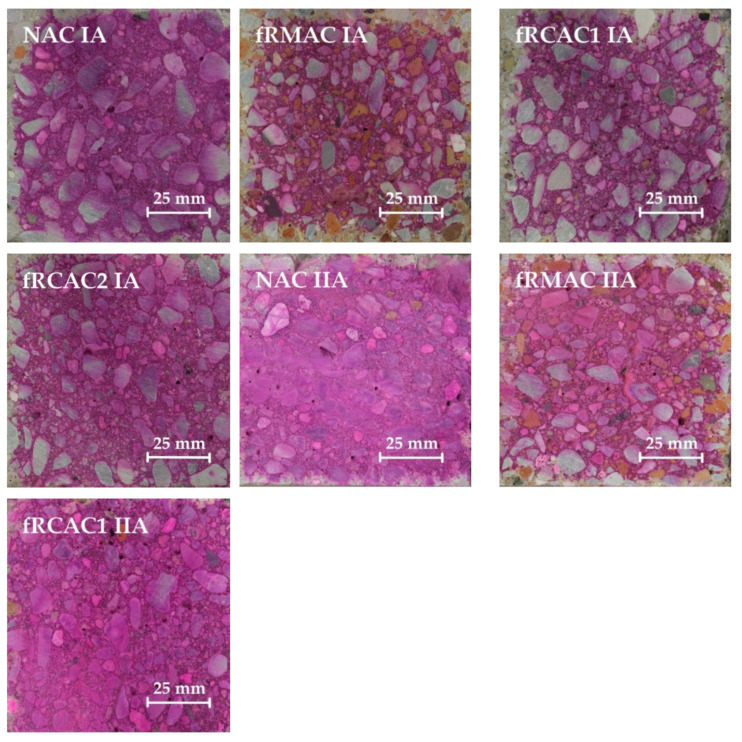

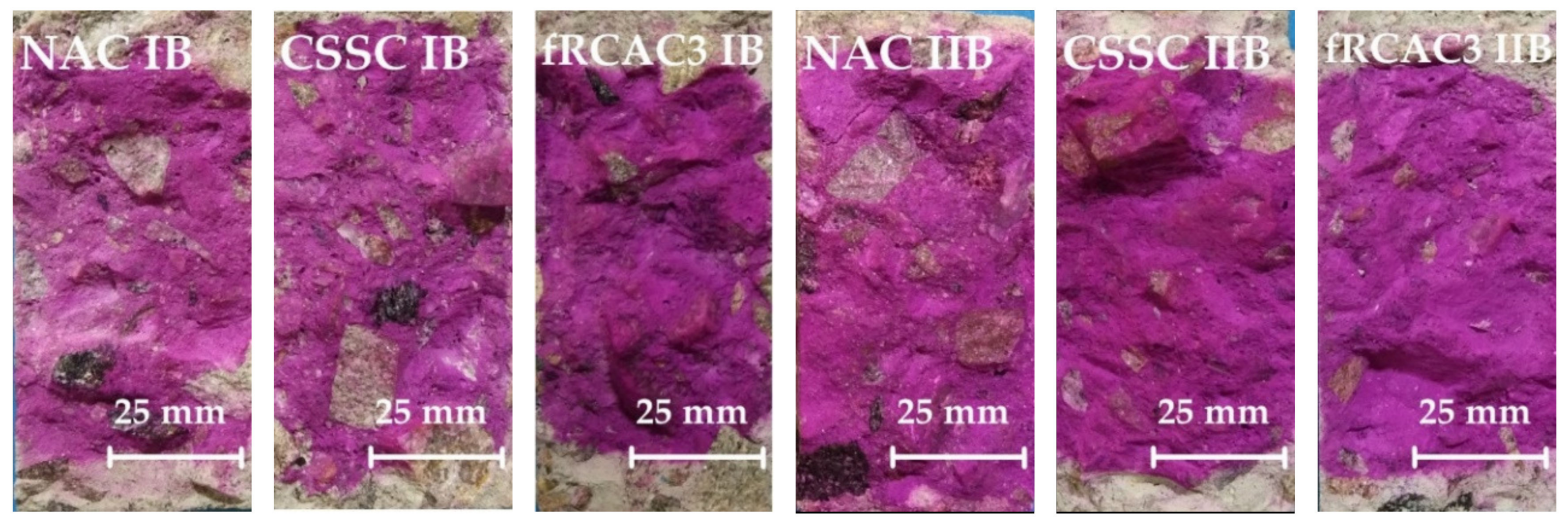
Carbonation depth of NAC and RAC.

**Figure 13:**
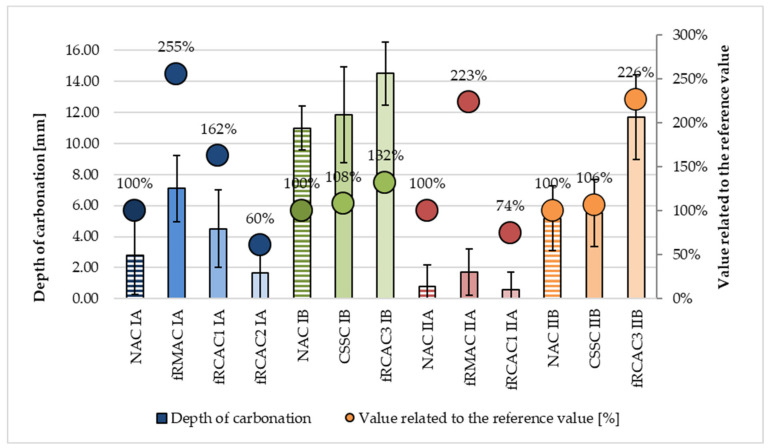
Comparison of carbonation depths of concrete containing fNA, fRCA, and fRMA with control mixtures.

Carbonation depth determines the quality of concrete’s protective cover over steel reinforcement bars. A poor carbonation resistance is bound to affect the service life of reinforced concrete structural elements [90]. In previous studies, the importance of using a reasonable amount of water was mentioned as essential for the carbonation resistance to carbonation of fRA concretes, especially when the amount of RFA exceeds 40%. It was reported that the higher amount of water unexpectedly did not improve the porosity of concrete but, on the contrary, worsened the carbonation resistance of fRAC. The optimal effective water-to-cement ratio was found to be essential for suitable resistance to carbonation of concrete [33,55]. This work confirmed the same observation. The depth of carbonation of concrete mixtures containing fNA, fRCA, and fRMA observed by both the teams is given in Table 8 and a comparison is presented in Figure 12 and Figure 13. The mixtures with a lower estimated effective water-to-cement ratio were seen to achieve better carbonation resistance. Moreover, the mixtures with a higher amount of cement seem to be more resistant to carbonation. The mixtures containing fRMA show a deeper penetration of CO_2_ into the concrete, probably caused by the high porosity fRMA. The increase in carbonation depth was 155% for fRMAC IA and 123% for fRMAC IIA. In the case of the Indian concrete mixtures, fRCAC3 IB was observed to have a higher depth of carbonation by 32% and 23% compared with NAC IB and CSSC IB, respectively. A significant increase in carbonation depth was observed in fRCAC3 IIB with respect to NAC IIB and CSSC IIB by 126% and 112%, respectively. The increase in carbonation depth may be attributed to the presence of high mortar content and more pores present in the fRCA concrete. Similar observations were found by [37,53,85]. On the contrary, fRCAC mixtures prepared by the Czech team achieved more favorable results in carbonation resistance, where increase in carbonation depth was observed in one mixture (fRCAC1 IA) only. However, from the point of view of previous studies carried out by the same research group [91,92], the negative influence of fRA is significantly lower than the impact of coarse RA.

## 6. Conclusions

In this study, the possibility of full replacement of natural sand in concrete by fine recycled aggregate was experimentally verified and discussed. This study was designed and implemented in the Czech Republic and India with the same research approach. The properties of fRA were examined by the validation of the physical and mechanical properties and durability of concrete containing fRA with a comparison between two different regions. The following conclusions are drawn.

- Differences were found in the properties of reclaimed sand originating from construction and demolition waste in two regions, which are probably caused by the parent concrete and differences in the recycling process. Moreover, the properties of concrete containing fRA slightly vary between the compared two regions, however, in both cases, the decline of properties in comparison with ordinary concrete is not limiting for finding satisfactory utilization.
- The density of fRA and, consequently, of fRAC decreases slightly compared with the natural sand and control mix, respectively. The water absorption of fRA and, consequently, of fRAC increases significantly compared to the natural sand control and control mix, respectively. On the contrary, the capillary water absorption decreases.
- The compressive strength mostly shows a slight decline; however, considering that the natural sand was fully replaced. For this reason, for future use of this material, the decline of compressive strength may be considered before a mixture design. The effect of the decline of the flexural strength is similar to compressive strength.
- In the case of modulus of elasticity, the highest decline in properties was found, which corresponds to previous studies, showing that the elastic modulus is the most decreased mechanical property of concrete with substitution by recycled aggregate in general. The static modulus of elasticity is the key characteristic of the material for the behavior of reinforced concrete structural elements because of its lower deflection of beams and slabs. For this reason, it is recommended to verify the strength and other technical requirements prior to using fRA as a full replacement of natural sand in reinforced concrete structures.
- In contrast, durability properties were not worsened significantly with fRA. The freeze–thaw resistance was completely satisfactory, and, furthermore, the carbonation resistance, although slightly affected, was not essential in terms of structural use since the concrete cover is usually 2 mm or higher. However, due to the significant decline of the modulus of elasticity, the use of fRAC as a complete replacement of natural sand for reinforcement concrete structures may need attention.

The novelty of this study was the comparison of the properties of fRA and fRAC in different regions in accord with SDG 17, which encourages partnerships to achieve the goal. The main objective of this study was to verify the transferability of practices and experiences of the substitution of sand in concrete by comparison of the results in two different regions with the same research approach. As mentioned in previous studies, the recycling technology and properties of parent concrete influence the properties of fRA, therefore, the material properties of fRA and fRAC were examined and compared in this investigation. Although minor differences in material properties were found between the two regions, the differences were not essential for future utilization, which will be more suitable for the complete replacement of sand in plain concrete structural elements, such as foundation structures, cement, concrete screed, etc., due to the local standards, the availability of the material, and the results of this investigation. Overall, this work represents the efforts in the direction of attaining a circular economy and thereby addressing the SDG 12.

## Figures and Tables

**Table 1:** The basic physical properties of each fraction of aggregates used for concrete mixtures.

RA Types	Grading (mm)	Finest Particles Content	Oven-Dried Particle Density		Water Absorption Capacity		Saturation Level
		f (%)	ρ_RD_ (kg/m^3^)	σ	WA_24_ (%)	σ	(%)
Natural aggregate (NA1)	0–4 4–8 8–16	0.3 0.3 0.4	2570 2530 2540	81 12 12	1.0 1.7 1.9	0.0 0.3 0.2	0.0 0.0 0.0
Natural aggregate (NA2)	0–4.75 4.75–10 10–20	0.0 0.0 0.0	2581 2670 2690	23 11 06	0.81 0.45 0.45	0.00 0.01 0.05	0.0 0.0 0.0
Crushed stone sand (CSS)	0–4.75	0.0	2596	83	2.78	0.18	0.0
Fine recycled masonry aggregate (fRMA)	0–4	1.0	2320	130	6.6	0.8	4.7
Fine recycled concrete aggregate (fRCA1)	0–4	0.6	2430	60	3.6	0.8	1.6
Fine recycled concrete aggregate (fRCA2)	0–4	2.0	2220	80	6.9	0.5	2.5
Fine recycled concrete aggregate (fRCA3)	0–4.75	0.0	2052	12	8.90	0.15	0.0

**Table 2:** The overview of test methods for aggregates.

Tests/Standards	The Czech Team	The Indian Team
Specific gravity/dry density	EN 1097-6	BIS 2386-3 (1963)
Water absorption of aggregates	EN 1097-6	BIS 2386-3 (1963)
Particle size distribution	EN 933-1	BIS 2386-1 (1963)

**Table 3:** Concrete mix proportion, per cubic meter.

Concrete Mixture	Cement	Water Mixing + Additional	W/C Ratio	SP	Natural Aggregate		Recycled Aggregate
					Fine	Coarse	Fine
	(kg/m^3^)	(kg/m^3^)	(-)	(kg/m^3^)	(kg/m^3^)	(kg/m^3^)	(kg/m^3^)
NAC IA	260	169 + 0	0.65	-	709	1130	0
fRMAC IA	260	169 + 18	0.72	-	0	766	971
fRCAC1 IA	260	169 + 17	0.71	-	0	949	843
fRCAC2 IA	260	169 + 34	0.78	-	0	946	773
NAC IB	260	130 + 12	0.55	2.3	813	1266	0
CSSC IB	260	130 + 28	0.61	3.1	813	1266	0
fRCAC3 IB	260	130 + 78	0.80	0.3	0	1266	813
NAC IIA	300	165 + 0	0.55	-	671	1167	0
fRMAC IIA	300	165 + 17	0.61	-	0	822	920
fRCAC1 IIA	300	165 +16	0.60	-	0	994	800
NAC IIB	320	144 + 12	0.49	1.6	779	1213	0
CSSC IIB	320	144 + 27	0.53	3.2	779	1213	0
fRCAC3 IIB	320	144 + 75	0.68	0.3	0	1213	779

**Table 4:** The overview of test methods for concrete samples.

Tests	Curing Period	The Czech Team		The Indian Team	
		Standards	Specimen Size	Standards	Specimen Size
	(Days)		(mm)	-	(mm)
Compressive strength	7, 28, 90, 180	EN 12390-3 (2003)	150 × 150 × 150	BIS 516 (1956)	150 × 150 × 150
Flexural strength	28	EN 12390-5 (2009)	100 × 100 × 400	BIS 516 (1956)	100 × 100 × 500
Static modulus of elasticity	28	EN 12390-13 (2014)	100 × 100 × 400	BIS 516 (1956)	150 diameter and 300 length
Dynamic modulus of elasticity	28	EN 12504-4 (2005)	100 × 100 × 400	BIS 13311-1 (1992)	150 diameter and 300 length
Carbonation	28	Inspired by ČSN EN 12390-12	100 × 100 × 200	RILEM CPC-18 (1988)	100 × 100 × 100
Freeze–thaw resistance	28	ČSN 73 1322 (1969)	100 × 100 × 400	Inspired by C666/C666M– 15 (2015)	100 × 100 × 500
Water absorption by immersion	28	Usual procedure of examination	100 × 100 × 100	Usual procedure of examination	100 × 100 × 100
Sorptivity	28	Inspired by ASTM C1585-20	100 × 100 × approx. 200	Inspired by ASTM C1585-20	100 × 100 × 100

**Table 5:** Density and water absorption of concrete mixtures.

Recycled Concretes	Dry Density		Water Absorption by Immersion		Water Absorption by Capillarity	
Designation	(kg/m^3^)	σ	(%)	σ	(kg/m^2^)	Σ
NAC IA	2301	18	5.89	0.35	2.31	0.30
fRMAC IA	2181	14	10.89	0.38	3.34	0.75
fRCAC1 IA	2276	11	7.68	0.25	1.98	0.22
fRCAC2 IA	2250	6	8.34	0.78	2.17	0.29
NAC IB	2373	5	6.30	0.66	3.83	0.05
CSSC IB	2391	74	8.80	2.88	4.53	0.25
fRCAC3 IB	2332	26	14.60	0.57	3.00	0.62
NAC IIA	2324	13	5.03	1.11	1.17	0.14
fRMAC IIA	2191	12	10.30	0.32	0.45	0.08
fRCAC1 IIA	2278	5	7.70	0.06	0.76	0.44
NAC IIB	2380	14	6.90	0.59	3.33	0.45
CSSC IIB	2193	11	8.30	0.87	1.93	0.48
fRCAC3 IIB	2188	21	13.20	0.31	1.70	0.36

**Table 6:** Average values and standard deviation of results of mechanical properties of concrete at age of 28 days.

Recycled Concrete Mixture	Compressive Strength		Flexural Strength		Static Modulus of Elasticity		Dynamic Modulus of Elasticity	
Designation	(MPa)	σ	(MPa)	σ	(GPa)	σ	(GPa)	σ
NAC IA	33.2	2.5	6.2	0.2	36.7 ^(1)^	1.4	38.2 ^(1)^	1.8
fRMAC IA	30.0	2.2	5.5	0.4	22.4 ^(1)^	1.0	27.3 ^(1)^	1.4
fRCAC1 IA	34.4	1.7	5.8	0.3	29.6 ^(1)^	0.4	34.5 ^(1)^	0.7
fRCAC2 IA	36.7	2.9	5.7	0.1	31.8 ^(1)^	1.2	35.4 ^(1)^	1.7
NAC IB	25.3	1.1	3.9	0.5	30.7 ^(2)^	0.0	41.2 ^(2)^	0.0
CSSC IB	30.5	0.7	4.3	0.7	24.4 ^(2)^	0.0	26.6 ^(2)^	0.0
FRCAC3 IB	22.5	1.2	6.5	0.6	25.3 ^(2)^	0.0	26.4 ^(2)^	0.0
NAC IIA	44.9	0.9	7.6	0.9	35.9 ^(1)^	0.5	38.2 ^(1)^	0.8
fRMAC IIA	38.0	0.9	6.8	0.6	25.3 ^(1)^	0.2	30.0 ^(1)^	0.9
fRCAC1 IIA	42.9	0.8	6.5	0.4	31.4 ^(1)^	1.0	35.7 ^(1)^	0.6
NAC IIB	35.8	0.6	4.3	0.1	33.2 ^(2)^	0.0	44.5 ^(2)^	0.0
CSSC IIB	35.8	0.9	5.3	1.1	29.3 ^(2)^	0.0	29.7 ^(2)^	0.0
FRCAC3 IIB	25.3	0.9	5.5	0.4	25.6 ^(2)^	0.0	32.6 ^(2)^	0.0

^(1)^ Examined on prismatic specimen 100 × 100 × 400 mm^3^.^. (2)^ Examined on cylindric specimen of 150 mm diameter and 300 mm length

**Table 7:** Average values of results of long-term compressive strength development, including standard deviation.

Age of Samples	7 Days		28 Days		90 Days		180 Days	
Designation	(MPa)	σ	(MPa)	σ	(MPa)	σ	(MPa)	Σ
NAC IA	-	-	33.2	2.5	36.5	3.0	41.5	1.5
fRMAC IA	-	-	30.0	2.2	33.2	1.5	34.2	2.0
fRCAC1 IA	-	-	34.4	1.7	37.0	3.0	38.1	1.5
fRCAC2 IA	-	-	36.7	2.9	40.8	1.1	41.8	2.7
NAC IB	23.6	0.5	25.3	1.1	31.3	0.6	-	-
CSSC IB	23.3	0.7	30.5	0.7	36.2	0.8	-	-
fRCAC3 IB	15.8	0.5	22.5	1.2	24.0	1.9	-	-
NAC IIA	-	-	44.9	0.9	47.4	3.3	52.1	0.7
fRMAC IIA	-	-	38.0	0.9	40.6	1.8	43.2	1.1
fRCAC1 IIA	-	-	42.9	0.8	42.4	4.1	48.2	0.5
NAC IIB	27.4	0.6	35.8	0.6	45.5	0.1	-	-
CSSC IIB	31.5	2.0	35.8	0.9	41.6	0.9	-	-
fRCAC3 IIB	15.3	0.6	25.3	0.9	26.4	1.9	-	-

**Table 8:** The comparison of the flexural strength before and after freezing–thawing, the frost resistance coefficient, and carbonation depth of concrete mixtures.

Recycled Concrete Mixture	Flexural Strength + Standard Deviation				Frost Resistance Coefficient	Freeze–Thaw Resistance	The Carbonation Depth
Designation	0 Cycles		100 Cycles		(-)	Cycles	(mm)
NAC IA	6.15	±0.22	6.87	±0.20	1.12	100	2.78 ^(1)^
fRMAC IA	5.53	±0.39	5.85	±0.40	1.06	100	7.10 ^(1)^
fRCAC1 IA	5.78	±0.30	6.57	±0.26	1.14	100	4.51 ^(1)^
fRCAC2 IA	5.65	±0.14	6.22	±0.27	1.10	100	1.68 ^(1)^
NAC IB	3.89	±0.46	3.44	±0.36	0.88	100	11.00 ^(2)^
CSSC IB	4.32	±0.72	3.35	±0.09	0.78	100	11.83 ^(2)^
fRCA3 IB	6.52	±0.61	3.15	±0.14	0.48	-	14.50 ^(2)^
NAC IIA	7.55	±0.87	7.80	±0.12	1.03	100	0.77 ^(1)^
fRMAC IIA	6.84	±0.60	6.78	±0.00	0.99	100	1.71 ^(1)^
fRCAC1 IIA	6.54	±0.44	6.73	±0.10	1.03	100	0.57 ^(1)^
NAC IIB	4.27	±0.08	4.00	±0.15	0.94	100	5.17 ^(2)^
CSSC IIB	5.25	±1.14	4.78	±0.64	0.91	100	5.50 ^(2)^
fRCA3 IIB	5.47	±0.41	3.36	±0.32	0.61	-	11.70 ^(2)^

^(1)^ Examined on prismatic specimen 100 × 100 × 200 mm. ^(2)^ Examined on specimen size 50 × 50 × 100 mm, longitudinal sides coated with epoxy paint.

**Table 9:** Dynamic modulus of elasticity after freezing and thawing.

Recycled Concretes		Dynamic Modulus of Elasticity (GPa) + Frost Resistance Coefficient (-)								Freeze–Thaw Resistance
Designation	0 Cycles	25 Cycles		50 Cycles		75 Cycles		100 Cycles		Cycles
NAC IA	37.6	36.4	0.97	36.5	0.97	35.8	0.95	36.9	0.98	100
fRMAC IA	19.7	17.0	0.86	18.4	0.94	17.6	0.89	19.3	0.98	100
fRCAC1 IA	35.1	32.3	0.92	30.9	0.88	32.4	0.92	31.3	0.89	100
fRCAC2 IA	37.3	34.3	0.92	33.3	0.89	33.2	0.89	33.1	0.89	100
NAC IB	29.31	-	-	-	-	-	-	25.94	0.89	100
CSSC IB	27.88	-	-	-	-	-	-	27.79	1.00	100
fRCA3 IB	23.89	-	-	-	-	-	-	30.78	1.29	100
NAC IIA	40.4	37.0	0.92	36.0	0.89	37.4	0.93	35.1	0.87	100
fRMAC IIA	31.6	28.4	0.90	25.9	0.82	29.8	0.94	28.0	0.88	100
fRCAC1 IIA	35.2	29.6	0.84	34.5	0.98	33.4	0.95	31.1	0.88	100
NAC IIB	25.37	-	-	-	-	-	-	21.17	0.83	100
CSSC IIB	18.91	-	-	-	-	-	-	24.42	1.29	100
fRCA3 IIB	24.84	-	-	-	-	-	-	22.59	0.91	100

## Data Availability

Not applicable.
